# Taxing Working Memory during Retrieval of Emotional Memories Does Not Reduce Memory Accessibility When Cued with Reminders

**DOI:** 10.3389/fpsyt.2015.00016

**Published:** 2015-02-12

**Authors:** Kevin van Schie, Iris M. Engelhard, Marcel A. van den Hout

**Affiliations:** ^1^Clinical and Health Psychology, Utrecht University, Utrecht, Netherlands

**Keywords:** dual taxation, memory accessibility, reaction time task, working memory, visual imagery

## Abstract

Earlier studies have shown that when individuals recall an emotional memory while simultaneously doing a demanding dual-task [e.g., playing Tetris, mental arithmetic, making eye movements (EM)], this reduces self-reported vividness and emotionality of the memory. These effects have been found up to 1 week later, but have largely been confined to self-report ratings. This study examined whether this dual-tasking intervention reduces memory performance (i.e., accessibility of emotional memories). Undergraduates (*N* = 60) studied word-image pairs and rated the retrieved image on vividness and emotionality when cued with the word. Then they viewed the cues and recalled the images with or without making EM. Finally, they re-rated the images on vividness and emotionality. Additionally, fragments from images from all conditions were presented and participants identified which fragment was paired earlier with which cue. Findings showed no effect of the dual-task manipulation on self-reported ratings and latency responses. Several possible explanations for the lack of effects are discussed, but the cued recall procedure in our experiment seems to explain the absence of effects best. The study demonstrates boundaries to the effects of the “dual-tasking” procedure.

## Introduction

Eye movement desensitization and reprocessing (EMDR) is an evidence-based treatment for posttraumatic stress disorder (1–3). In EMDR, patients are asked to recall traumatic memories while they simultaneously make eye movements (EM). Although EMDR’s scientific and practical value was questioned at first (4), EMDR has proven to be as effective as trauma-based cognitive-behavioral therapy [For meta-analyses, see, e.g., Ref. (5, 6)].

For a long time, it has been controversial whether making EM while recalling the distressing memory added anything to the procedure (7). A recent meta-analysis of clinical and laboratory studies on the role of EM has shown that EM during recall of negative memories do have additional effects (8). For laboratory studies, this additive effect is evident by larger reductions in subjective vividness and emotionality ratings of the distressing memory for recall + EM conditions, compared to a control condition in which participants merely recall the memory (i.e., recall only). Clinical studies have mainly found effects of EM on Subjective Units of Distress. There are some findings of EM effects on Subjective Units of Distress combined with symptom measures, such as the Impact of Events Scale [see Ref. (8)].

The effectiveness of EM in EMDR can be explained by dual taxation of the limited resources of working memory [WM, e.g., Ref. (9)]. This dual taxation takes place when an individual recalls a distressing memory while also performing a secondary task, such as making EM, mental arithmetic, or drawing complex figures. When individuals perform the secondary task and simultaneously recall a memory, both tasks compete for scarce WM resources. During this competition, the distressing memory cannot be retrieved completely (i.e., gets blurred) and is stored as a blurred memory after this competition. As a consequence, the blurred memory will be retrieved during future recalls. Presently, a body of evidence supports the WM hypothesis [e.g., Ref. (10–12); for review, see Ref. (13)]. The vast majority of studies have shown that dual taxation of WM blurs *autobiographical* memories [see Ref. (8, 13)], with a few exceptions, such as pictures (9, 14) and a film clip (15).

Most studies on the effects of recall + EM focused on changes in the subjective ratings of the experienced memory [e.g., Ref. (10, 16, 17)]. Therefore, the effects of EM in these studies could still, in part, be the effect of demand characteristics; inferences participants make based on what they think the researcher expects (11). Thus, it remains unclear whether changes in subjective ratings are the result of an experimental manipulation *per se* or of unconscious or conscious alterations in the participants’ behavior to fit the hypothesis. Gunter and Bodner (11) showed that simultaneous recall + EM reduced subjective ratings, while making EM *after* memory recall did not produce these reductions. This seems to forestall the conclusion that demand characteristics fulfill a large role in the effects of EM, because if they did, both conditions should yield similar results. Nevertheless, objective memory measures could preclude the demand characteristics account even further.

To date, however, little attention has been devoted to objectively measure memory accessibility. Yet, WM theory predicts blurring of memories that are recalled under dual taxation conditions. This reduces its accessibility, because it is harder to access a blurred memory. Van den Hout et al. (18) attempted to objectively measure alterations in memory vividness with a reaction time (RT) task. In their experiment, participants studied two “neutral” pictures, though the pictures were not rated for emotional valence. One picture was followed by recall + EM and the other was followed by recall only. In the RT task, participants performed old/new recognition on cut-outs taken from both pictures and cut-outs from never studied pictures. The rationale for using cut-outs was that comparing a picture cut-out with a blurred memory of that same picture takes more time, than a non-blurred memory. Van den Hout et al. (18) showed that in the Recall + EM condition – but not in the recall only condition – a reduction in vividness ratings was accompanied by an increase in RTs: participants were slower in deciding whether they had seen the fragments before. Therefore, RTs seem suitable as an objective behavioral measure to assess the effects of memory blurring by EM. Notably, in a different paper (14), the question arose whether the allegedly neutral pictures from Van den Hout et al. (18) were truly neutral. As an annex, in the discussion of the later paper, the two pictures of Van den Hout et al. (18) were rated and it was observed that the pictures were hedonically positive. It is not self-evident that the effect of reduced accessibility of emotionally positive stimuli generalizes to negative materials.

While traumatic memories may seem to occur out of the blue, they are often activated by environmental cues (19). Therefore, the blurring of emotional memories needs to be placed in perspective. According to cognitive theories about posttraumatic stress disorder, traumatic memories are not contained in a vacuum, they can be activated by external cues. For instance, Brewin (20) argued that certain cues reminiscent of the traumatic experience can activate memory presentations that are otherwise inaccessible. Ehlers and Clark (21) make a comparable statement, namely, that involuntary intrusive visual memories are triggered by stimuli that are temporally associated with the trauma, but are not strongly semantically related to the event. Through an associative learning process these stimuli and trauma become connected, and as a consequence the stimulus becomes a warning signal: a stimulus that signals imminent danger (22).

Though it is apparent that traumatic memories are not isolated memories, it is currently unclear whether the effects of EM (i.e., reductions in subjective ratings and in memory accessibility) will be found when a reminder cue is presented. On the one hand, it is easier to remember episodic information in cued recall than in non-cued recall [e.g., Ref. (23)]. On the other hand, cues are frequently not uniquely encoded with one memory, which may make it difficult to recall a specific memory instantly. Furthermore, reduced subjective ratings do not necessarily have to co-occur with reduced accessibility, because declarative memory and memory for learning associations (i.e., conditioning) can be dissociated (24). To illustrate this point, patients with posttraumatic stress disorder may display trouble with intentional recall of aspects of the trauma memory, while cue-driven triggers lead to intrusive re-experiencing of that memory (21).

Inaccessibility of episodic memories has frequently been studied with the Think/No-Think paradigm (25). In this task, participants first learn cue-target word pairs. They then repeatedly recall the target for “think items” when seeing the cue, or stop target retrieval for “no-think items.” For one-third of the items (baseline items), there is no recall or retrieval-stopping. Afterwards, memory for all pairs is assessed and generally a part of the cued no-think items – compared to baseline items – have become inaccessible [see Ref. (26) for a review]. Evidently, the accessibility of cued episodic memories can be affected.

The main aim of the current study was to replicate and extend the study of Van den Hout et al. (18) to negative memories. We tested whether recall + EM affect the accessibility of associative emotionally *negative* memory representations. Studies on the effects of dual taxation on memory typically use negative autobiographical memories. However, given that autobiographical memories are by their very nature hard to control and that positive effects have also been found with self-irrelevant memories for pictures shown in the laboratory [e.g., Ref. (18)], we decided to use memories of aversive pictures instead. We examined whether accessibility of emotional memories is affected when those memories are activated via *reminder cues*. We used these reminder cues as an experimental analog of the often cued nature of trauma intrusions. Additionally, we attempted to *objectively* measure memory blurring with a response latency task. To achieve our aim, we adapted the frequently used Eye Movements Task by incorporating elements from the think/no-think paradigm and reasoned that associative accessibility, measured by RT following a reminder cue, should be reduced for recall + EM compared to a recall only or to no-presentation control. Additionally, parallel to this RT reduction, we expect memory blurring in terms of reductions in self rated vividness and emotionality of the emotionally negative memory.

### Ethics statement

The research reported in this article involved healthy human participants, and did not utilize any invasive techniques, substance administration, or psychological manipulations. It was conducted according to the principles expressed in the Declaration of Helsinki. The sample size was set before data collection and written informed consent of each participant was obtained. In giving consent, participants indicated to have read and to have agreed with both the rules regarding participation and proper (laboratory) behavior, and the researchers’ commitments and privacy policy. They were also informed that they would be able to stop participating in the experiment whenever they wanted to do so. After consent, participants were randomly allocated to conditions and all gathered data were analyzed anonymously. Afterwards participants were debriefed.

## Materials and Methods

### Participants

Sixty-two undergraduates of Utrecht University (*M* = 22.12 years, SD = 3.16; 45 females, 17 males) participated for course credit or financial reimbursement. Two participants were excluded (one because of sudden illness, another because of EMDR knowledge prior to the experiment), resulting in a sample of 60 participants.

### Materials

#### Words

In this paradigm, participants studied word-image association pairs that were divided over recall + EM, recall only, and control. For these pairs, 12 neutral Dutch words and two filler words (bean, caterpillar, chip, clock, gate, hawk, iron, mill, nail, plum, reptile, spleen, stamp, and wind) with moderate levels of arousal were selected from Moors et al. (27) (*M*_valence_ = 4.08, SD = 0.15; *M*_arousal_ = 3.59, SD = 0.41; words were rated on a scale from 1 *very negative/passive* to 7 *very positive/active*). Filler were used as buffers at the beginning and end of lists to avoid primacy and recency effects. Importantly, the three experimental conditions were matched on ratings of valence and arousal, as well as on ratings of word length, word frequency, power, and age of acquisition. The latter two referred to the extent to which a word is submissive/dominant, and to the estimated age a word was first learned.

We used neutral words as a model of cued trauma recall. Frequently, objects or situations that are associated with trauma – because of their temporal proximity – are of neutral valence (e.g., a bank in case of a bank robbery). A second reason to use neutral words instead of negative words was to avoid inter-pair associations as much as possible (i.e., associations other than those between the word and image of a pair). Because negative materials stem from a small number of categories (e.g., death, disease), they are related to each other quickly. Since, the use of negative images was crucial, use of negative words would rapidly increase inter-pair associations. Moreover, we made sure cue words were not related to other cue words in the stimulus set with the association database from the University of Leuven: www.kuleuven.be/semlab.

#### Images

In a pilot study, participants (*N* = 24) rated 52 potentially neutral and 52 potentially negatively valenced images from IAPS (28) and Google Image. Pictures were rated with the Self-Assessment Manikin [SAM; (28)] on a nine-point rating scale, where nine represents a high score on each dimension, and one represents a low score. From the pilot data, 12 images (and two filler images) with the lowest valence and highest arousal ratings were selected (*M*_valence_ = 2.29, SD = 0.53; *M*_arousal_ = 6.2, SD = 0.85). Five IAPS images were selected (2053, 6313, 6821, 9433, and 9911) and seven Google Image Pictures depicting a bullfighter attacked by a bull; an anorexic woman looking in the mirror; masked soldiers carrying guns and explosives; the hanging of two men; elephants killed by poachers; and a man who set himself on fire. Images had a landscape orientation and were of the same size (500 × 375 pixels). For the final test, each image was divided into four equally sized cut-outs (250 × 187 pixels).

#### Visual analog scale

Participants rated the dependent variables vividness, emotionality, difficulty to retrieve the memory of the target scene, and degree of confidence in their decision (“choice confidence”; see end of Section “Response Latency Task”) on a visual analog scale (VAS) from 0 (*not vivid/emotional/difficult/certain at all*) to 100 (*very vivid/emotional/difficult/certain*). Choice confidence was added as a novel outcome measure, because correct target image identification in forced choice can be independent of choice confidence. Decreases in confidence are an extremely robust finding of research on obsessive–compulsive disorder in our laboratory [e.g., Ref. (29)].

#### Response latency task

Four cut-outs were taken from each picture (48 cut-outs in total). Participants were asked to identify which cut-out had been paired earlier with the cue word presented on screen. They consecutively rated their choice confidence. The cue was displayed for 1000 ms followed by four cut-outs presented in four quadrants around the cue. The correct cut-out belonging to the target image had to be selected within 4000 ms by pressing the button on the numerical keypad that corresponded with the cut-out’s location on screen. To avoid learning from novelty elimination, three other cut-outs displayed parts from images that had been used as targets for other cues. There was one pseudo-randomized order set, wherein the total serial position for each condition was identical, and no more than two cues from the same condition were displayed consecutively. Response latency was measured as dependent variable. Additionally, participants rated how confident they were their answer was correct (400 ms intertrial interval; ITI).

#### Post-experimental questions

Participants rated on paper-and-pencil VAS to what extent they were compliant with and had been able to follow the instructions, which was used as a manipulation check. For recall + EM and recall only they rated to what extent they made EM, were able to recall the cued target memory, and how vivid and detailed that memory was.

### Procedure

#### Learning phase

Initially, all cue-target pairs – consisting of a word and image – were presented in the middle of a black screen for 8000 ms followed by 400 ms ITI. The display time was taken from Depue, Banich, and Curran (30) and was doubled, because participants were instructed not only to associate cue and target but also to be able to recall the target image as complete and detailed as possible when seeing the cue. Based on a pilot, participants indicated that this display time was sufficient to comply with instructions. Next, participants saw the cue word for 1000 ms and were instructed to select the correct target image for each cue from four image options presented in four quadrants around the cue. Three images displayed scenes that were targets of other cues. Cue and image options disappeared after 4000 ms or after target selection. The maximum decision time was based on a pilot, in which all decisions made by participants were within 4000 ms. The correct target was highlighted by presenting a green rectangle around the correct target for 2000 ms followed by 400 ms ITI (31) regardless of the participant’s answer. When participants correctly selected 11 out of 12 experimental targets, they proceeded to the learning test. They had up to six list repetitions to achieve this criterion. All participants reached this criterion within six repetitions.

#### Learning test

In the learning test (pre-test), participants were presented with a cue word and retrieved the accompanying target as vividly and detailed as possible, and pressed the spacebar when they did. They then rated the memory of the retrieved target on vividness, emotionality, and difficulty of retrieval. Next, the cue was presented a second time for 1000 ms, and the participant had to select the correct target from four scenes within 4000 ms. Contrary to the learning phase, no feedback was provided. After each decision, participants rated how confident they were their answer was correct (400 ms ITI). After participants completed the learning test for all cues, they proceeded to the EM phase.

#### Eye movement phase

Participants were instructed to retrieve and visualize the target as vividly and detailed as possible after a cue was presented. For one-third of the cues, participants were instructed to simultaneously follow a dot of 20 pixel that moved laterally with their eyes (1 Hz frequency and 461 pixel amplitude) for 4 intervals of 24 s separated by 5-s breaks (“Recall + EM”). For another third of the cues, the same procedure was used except that participants did not perform a secondary task, but simply looked at the center of the screen while thinking of the target (“Recall Only”). The final third of the cues were not presented in this phase and served as “natural decay” control condition. The duration of the experimental manipulations was identical to previous studies [see Ref. (13)]. In total, eight cues were presented in this phase and no more than two cues with the same instruction were given in a row. Word pairs were rotated through conditions over participants. After the 4 × 24 s of the eighth cue, participants continued with the final test phase.

#### Final test phase

Memory for all experimental items was assessed in the final test (post-test). Participants were presented with the cue and were instructed to retrieve the memory of the image associated with the target. They then rated vividness and emotionality of the memory of the retrieved target, and did this for all cues before continuing to latency response task. After the latency response task, participants filled-out the post-experimental questionnaire.

## Results

Data with more than three SD from the mean were corrected. (Results for data with and without outlier correction were comparable.) Moreover, in order to retain sufficient power, slight violations of sphericity were corrected with Greenhouse–Geisser (0.70 ≥ ε < 0.75) or Huynh–Feldt corrections (ε ≥ 0.75). In case of severe violations (ε < 0.70) a multivariate test statistic (Pillai–Bartlett trace; *V*) is reported. Analyses were performed only on pairs for which participants recalled the target on the final learning test. Table 1 presents means and SD of the self-report ratings for the three conditions.

**Table 1:** **Means and SD (in parentheses) of difficulty, confidence, vividness, and emotionality ratings for the recall + EM, recall only, and control conditions**.

	Difficulty	Confidence	Vividness		Emotionality	
			Pre	Post	Pre	Post
Recall + EM	29.85 (16.82)	89.90 (11.6)	72.30 (14.22)	73.90 (16.59)	55.16 (16.23)	53.99 (19.04)
Recall only	31.21 (15.68)	89.05 (10.88)	70.77 (14.25)	73.59 (14.74)	51.05 (20.13)	53.07 (17.46)
Control	33.78 (17.93)	88.90 (12.05)	70.74 (14.72)	71.27 (15.28)	50.43 (19.74)	53.16 (19.68)

### Manipulation checks

An analysis of variance (ANOVA) revealed that there were no differences between conditions on difficulty of retrieval, *F*(2, 118) = 1.56, *p* = 0.21, $\eta_{p}^{2}=0.03,$ indicating comparable levels of recall before entering the EM phase.

On the post-experimental questions, participants indicated that they frequently made EM during recall + EM (*M* = 83.77, SD = 14.58), and rarely during recall only (*M* = 13.87, SD = 18.68), *t*(59) = 18.79, *p* < 0.001, *d* = 4.2. They were better able to recall the memory of the image during recall only (*M* = 80.63, SD = 15.40) compared to recall + EM (*M* = 64.78, SD = 24.36), *t*(59) = 4.91, *p* < 0.001, *d* = 0.78. Additionally, the recalled image was more vivid and detailed during recall only (*M* = 72.35, SD = 18.50) compared to recall + EM (*M* = 52.25, SD = 24.15), *t*(59) = 6.56, *p* < 0.001, *d* = 0.96.

### Vividness

A 2 (Pre, Post) × 3 (recall + EM, recall only, control) ANOVA showed no significant main or interaction effects for vividness ratings, *F*s < 1.90, *p*s > 0.17, $\eta_{p}^{2}<0.04.$

### Emotionality

A 2 (Pre, Post) × 3 (recall + EM, recall only, control) ANOVA did not reveal significant main or interaction effects, *F*s < 2.23, *p*s > 0.13, $\eta_{p}^{2}<0.04.$

### Confidence

Because confidence ratings related to complete images before the experimental manipulation and to partial images after the manipulation, the former were entered as covariates in an ANCOVA. Pre-manipulation confidence ratings for recall + EM, *F*(1, 56) = 17.38, *p* < 0.001, $\eta_{p}^{2}=0.24,$ and for control, *F*(1, 56) = 11.87, *p* = 0.001, $\eta_{p}^{2}=0.18,$ related significantly to post-manipulation confidence ratings. There was no relation between pre-manipulation recall only scores and post-manipulation ratings, *F* < 1. The main analysis of condition on post-manipulation ratings showed no effect when controlling for the pre-experimental confidence ratings, *F*(1.672, 93.641) = 0.17, *p* = 0.80, $\eta_{p}^{2}=0.003$ (Huynh–Feldt correction).

### Response latencies

Before analyses, response latencies were log transformed because of natural skewness in scores. Similar to confidence ratings, response latencies related to complete images before the experimental manipulation and to partial images after the manipulation. Therefore, pre-manipulation response latencies were entered as covariates in an ANCOVA. Pre-manipulation response latencies for recall only, *F*(1, 56) = 6.70, *p* = 0.012, $\eta_{p}^{2}=0.11,$ related significantly to post-manipulation response latencies. Other pre-manipulation response latencies did not, *F*s < 3.10, *p*s > 0.08, $\eta_{p}^{2}<0.06.$ The main analysis for post-manipulation response latencies, though, did not reach significance when controlling for pre response latencies, *F*(2, 112) = 0.7, *p* = 0.5, $\eta_{p}^{2}=0.01.$ Table 2 presents means and SD.

**Table 2:** **Means and SD (in parentheses) of response latencies and accuracy for recall + EM, recall only, and control conditions**.

	Response latencies	Accuracy
Recall + EM	1268.7 (310)	0.97 (0.06)
Recall only	1292.4 (383.8)	0.96 (0.07)
Control	1280.9 (324.6)	0.97 (0.06)

### Accuracy

Participants needed, on average, 1.77 (SD = 1.30) repetitions to achieve the learning criterion. Because analyses were performed only on pairs for which participants recalled the target on the final learning test, pre-manipulation scores for accuracy reached the ceiling (100% for all conditions). Therefore, for accuracy, an ANOVA was performed on post-manipulation scores only (see Table 2). Participants did not differ in their accuracy to select the correct target images for the different conditions, *F*(2, 118) = 0.45, *p* = 0.64, $\eta_{p}^{2}=0.007.$

## Discussion

The aim of this study was to test whether recall + EM affect the accessibility of associative memory representations when emotionally negative materials are used. In this extended replication of Van den Hout et al. (18), we found no blurring of the emotional memory representations for recall + EM compared to recall only or no intervention. This was reflected in the absence of any effects on subjective ratings of vividness and emotionality, and also in objective measures of memory accessibility, specifically latency responses. Participants reported they had complied with the instruction to make EM. They also reported that mental images were less vivid, less detailed, and more difficult to retrieve *during* the intervention. However, this seems trivial, because these effects did not persist at the post-test.

The data show no reductions in memory accessibility, but they do not necessarily falsify WM theory as an explanation for the effects of dual taxation. The absence of effects may be a consequence of our materials. Could the data have been different if self-relevant or less negative pictures would have been used? The vast majority of earlier studies on the effects of demanding secondary tasks have used personally relevant, autobiographical memories [e.g., Ref. (10, 11, 32)], while our study used novel emotional images. These images could have lacked the potential to elicit sufficient levels of arousal, which may be necessary in the dual-tasking procedure to reduce vividness (Littel et al., submitted). Van den Hout et al. (14) showed that only negative autobiographical memories – which are associated with transient levels of arousal – were reduced in their vividness ratings, while neutral memories were not. This suggests arousal is a prerequisite for (re-)encoding of memories after dual taxation. Although our materials were not negative autobiographical memories, they were, however, thoroughly piloted and showed sufficient arousal during the pilot and on pre-test measures. Moreover, other studies have used non-idiosyncratic materials and showed effects for recall + EM compared to recall only [e.g., Ref. (15)] and specifically showed that effects can be found for materials that are neither autobiographical nor self-relevant (9, 18). It therefore seems unlikely that intrinsic qualities of our novel images *per se* explain the absence of effects.

The tasks we used may also have limited the effects of dual taxation. This study used a latency response task that was based on a similar task in Van den Hout et al. (18). In their task, participants were instructed to react as fast and accurately as possible, and had to decide whether a cut-out was old or new. In our experiment, participants received comparable instructions. Yet, they did not make an old-new judgment, but a source judgment: they had to indicate which of the four displayed images was the cue’s target. Though these tasks look similar, they probably draw on different types of recognition: old-new recognition and source recognition. Old-new recognition can generally be performed at lower levels of item differentiation than source decisions (33). As a consequence, slightly blurred recall + EM pairs may show effects for old-new decisions compared to recall only pairs, but not for source decisions. It is possible that successful source memory differentiation does occur when pairs from the recall + EM condition differ more in the level of memory blurring from the recall only or no-presentation control conditions. Theoretically, it may be possible to find differences between conditions when an old-new recognition task is used instead of a source recognition task.

A different explanation may also be found in the response latency task, specifically in the distractors that were used as targets in previous trials. During the final part of the experiment, participants had to select the correct target out of four cut-out images. Here, the use of distractors that have been used as targets in previous trials may cause response inhibition, and related response delays. Alternatively, it is also possible that the blurring effects were abolished, when participants specifically saw recall + EM images as distractors in earlier trials, and in later trials saw these same images as targets. As a consequence, the image could have been reinstated in full and any condition effects were abolished as well. This does not, however, explain why there were no effects on any of the subjective ratings, which preceded the response latency task. Though, it might be possible that the effects of recall + EM in our design were subtle and only detectable with RT, but that this effect was abolished by how our response latency task was designed. Furthermore, emotional interference may have played a role, because participants had to select one out of four highly unpleasant images, which may have caused response delays that are not due to simple blurring or retrieval delay. Indeed, it cannot be ruled out that there might be a floor effect in that the delay is beyond the critical point where retrieval differences can be found.

Though several plausible explanations for the lack of effects can be found in the response latency task, it still leaves unanswered why subjective ratings of the images did not change, because these ratings preceded the latency response task, and were recalled without seeing any of the targets from other pairs. Perhaps cued recall of the to-be-recalled material influenced the effectiveness of dual taxation. On the one hand, cued recall could facilitate episodic memory retrieval compared to non-cued recall (23), and thus should allow the participant to vividly retrieve the associated target image. This is reflected in relatively low pre-test scores for self-assessed difficulty of retrieval and high scores for vividness. Subsequent retrieval of a vividly cued image should therefore be blurred as a consequence of dual taxation. This, however, did not happen. On the other hand, even though participants quickly learned the associations – which hints at strong relationships between cue and target – these may simply not have been strong enough. We used neutral words referring to objects that people may encounter frequently in daily life and thus could be linked to various situations. Although the association between the cue and target is novel and recent, it is unlikely that the cue’s path exclusively leads to the target. Therefore, after images were cued in a recall + EM trial, participants may not have thought of the target image all the time, but of other images, objects, or words. As a consequence, the target may not have been sufficiently blurred and reductions in self-reported vividness and emotionality may not have been experienced for recall + EM. Hence, generally cued recall may ameliorate memory, but not when a multipath cue needs to prompt one specific target for prolonged periods of time. Additionally, this might also explain why there were no latency response effects. If cues did not elicit specific and continuous target retrieval, then differential item blurring and successful source recognition could not have occurred.

Provided that cued recall was primarily responsible for the lack of effects, the question ensues whether dual taxation is able to affect associative memory networks. A review of earlier studies showed that WM taxation, specifically EM, is able to reduce subjective ratings of emotional memories when those memories were recalled immediately after the intervention (8). The current study showed that these memories were not changed subjectively or objectively when cued with a memory reminder. Perhaps this limitation signals a boundary condition for this paradigm and limits the robustness of the dual taxation paradigm. Until now, Van den Hout et al. (18) conducted the only study that found effects of the EM intervention on objective measures of memory accessibility, and it cannot be ruled out that this represents a chance finding. It should be noted that other studies using objective measures of memory valence or emotionality have shown effects after dual taxation, such as eye blink startle reflex diminution (34), reduced heart rate variability (35), and decreased electrodermal responses (36), but these objective measures primarily related to arousal levels and not memory accessibility *per se*.

If it does, however, signal a limitation of the dual taxation paradigm, then memory change under dual taxation conditions may require a specific form of recall. This would imply that a memory must be directly recalled as opposed to memory recall that is initially cued by reminders. The former has been frequently used in previous work [e.g., Ref. (14)]. Interestingly, this does not preclude the possibility of finding effects with cued recall. Cued recall *after* dual taxation may still lead to reductions in subjective ratings, but only if the memory is recalled directly *during* dual taxation. This does, however, seem to contradict predictions from current trauma theories (20, 21), which state that encountering cues associated with an emotional memory could instantly trigger vivid intrusions of that memory. In the current study, cues probably elicited retrieval of the target memory, but most likely only briefly.

The lack of effect of cued recall also has implications for associative network theories, in which connectivity between different representations is paramount. A central tenet of network theories is modifiability of the network’s structure after activation [e.g., Ref. (37)]. It is possible that small networks (e.g., two nodes representing only stimulus characteristics, such as gate – car accident) are difficult to modify. Larger, more ecologically valid networks typically also contain elements regarding responses (“panic”) or meanings (“I am helpless”), next to mere stimulus relations. Perhaps these former relations are an inherent changeable part of the associative network, and also a part that does not need to be targeted directly. It is possible that stimulus characteristics change during dual taxation, and affect response and meaning elements, which changes how a person feels or thinks about an event. As a consequence, change in subjective experiences may be difficult to accomplish in smaller, laboratory created networks because these lack elements of meaning.

In sum, we found that EM during recall did not blur emotional memory representations measured by subjective or objective measures of memory accessibility. Response inhibition and emotional interference do not seem able to explain all the effects. Cued recall, on the other hand may; it may not have been potent enough to elicit specific and continuous target retrieval for differential item blurring to occur. Although memory effects following EM were not observed, it is unlikely – given the substantial body of evidence – that reductions in self-reported ratings are a chance discovery. Changes in objective measures of memory accessibility therefore still need to pass the critical test of replication.

## Conflict of Interest Statement

The authors declare that the research was conducted in the absence of any commercial or financial relationships that could be construed as a potential conflict of interest.

## Abbreviations

EM, eye movements; EMDR, eye movement desensitization and reprocessing; WM, working memory.
